# Septins in kidney: A territory little explored

**DOI:** 10.1002/cm.21477

**Published:** 2018-08-17

**Authors:** Anita A. Wasik, Surjya N. Dash, Sanna Lehtonen

**Affiliations:** ^1^ Department of Pathology University of Helsinki Helsinki Finland

**Keywords:** ciliogenesis, cytoskeleton, kidney, septins, zebrafish

## Abstract

Septins are a conserved family of GTP‐binding proteins that assemble into cytoskeletal filaments to function in a highly sophisticated and physiologically regulated manner. Originally septins were discovered in the budding yeast as membrane‐associated filaments that affect cell polarity and cytokinesis. In the last decades, much progress has been made in understanding the biochemical properties and cell biological functions of septins. In line with this, mammalian septins have been shown to be involved in various cellular processes, including regulation of cell polarity, cytoskeletal organization, vesicle trafficking, ciliogenesis, and cell–pathogen interactions. A growing number of studies have shown that septins play important roles in tissue and organ development and physiology; yet, little is known about their role in the kidney. In the following review, we discuss the structure and functions of septins in general and summarize the evidence for their presence and roles in the kidney.

## 1 SEPTINS, A FAMILY OF CONSERVED CYTOSKELETAL GTPASES

### 1.1 Structure of septins

Septins are found in fungi, protists and animals but not in plants (Nishihama, Onishi, & Pringle, 2011; Pan, Malmberg, & Momany, 2007). The number of septins varies between organisms, and for example, 13 septins have been identified from human (*Homo sapiens; SEPT1–12*, *SEPT14, SEPT13* is a pseudogene; Hall & Russell, 2004; Ihara et al., 2005). The human septins are classified into four groups based on sequence homology: SEPT2 (SEPT1, 2, 4, and 5), SEPT3 (SEPT3, 9 and 12), SEPT6 (SEPT6, 8, 10, 11, and 14), and SEPT7 (SEPT7) groups. Many septins undergo alternative splicing generating a great number of isoforms further increasing the complexity of the septin family (Hall & Russell, 2004).

**Figure 1:**
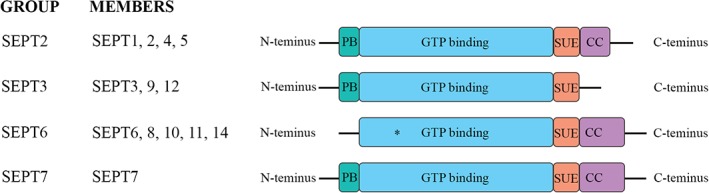
Schematic structure of septins. CC = coiled‐coil domain; PB = polybasic region; SUE = septin‐unique element. Asterisk indicates the lack of the Thr78 residue in the SEPT6 group, which prevents them from hydrolyzing GTP to GDP [Color figure can be viewed at wileyonlinelibrary.com]

The full‐length septin cDNAs encode polypeptides of 30–65 kDa, which typically consist of four conserved domains: a polybasic region, a GTP‐binding domain, a septin‐unique element, and a coiled‐coil domain (except for the SEPT3 subgroup) (Mostowy & Cossart, 2012) (Figure 1). The GTP‐binding domain mediates the binding and hydrolysis of GTP (Mostowy & Cossart, 2012). Septins belonging to the SEPT6 group lack the key Thr residue (Thr78), which prevents them from hydrolyzing GTP to GDP (Mostowy & Cossart, 2012). The polybasic region mediates the association of septins with the plasma membrane, and the coiled‐coil domain plays a role in protein–protein interactions. SEPT4, SEPT8, and SEPT9 contain a proline‐rich domain at the N‐terminus that binds SH3 domains and mediates protein–protein interactions (Figure 1) (Hall & Russell, 2004). The proline‐rich and coiled‐coil domains vary in length and amino acid composition (Hall & Russell, 2004).

Septins localize to the cleavage furrow in dividing cells and along actin stress fibers in interphase cells. Septins assemble into homo‐oligomers in vitro and hetero‐oligomeric polymers in vitro and in vivo (Fung, Dai, & Trimble, 2014; Joberty et al., 2001; Kinoshita, 2003; Nagata, Asano, Nozawa, & Inagaki, 2004). The polymers, in turn, are arranged in tandem arrays to form 7‐ to 9‐nm thick filaments and can also arrange into bundles, such as rings and coils (Kinoshita, 2003). The assembly of septin complexes and filaments is regulated by several factors, including GTP‐binding and hydrolysis and potentially also the binding partners (Sirajuddin, Farkasovsky, Zent, & Wittinghofer, 2009). Moreover, membrane lipids, actin cytoskeleton, or microtubules have an impact on polymerization of septins into filaments (Kinoshita, Field, Coughlin, Straight, & Mitchison, 2002; Nagata et al., 2003).

### 1.2 Function of septins

Originally, septins were discovered in the budding yeast *Saccharomyces cerevisiae* as membrane‐associated filaments that affect cell polarity and are required for mother–daughter separation during cytokinesis (Haarer & Pringle, 1987; Hartwell, 1971). In addition, septins associate with the plasma membrane by directly binding to phospholipids through their polybasic domains (Zhang et al., 1999) and participate in limiting the diffusion of endoplasmic reticulum (ER) and nuclear envelope proteins by recruiting membrane‐associated factors (Luedeke et al., 2005). Indeed, a septin barrier inhibits the movement of ER membrane proteins from traversing the mother–bud neck (Luedeke et al., 2005). The role of septins in the recruitment or regulation of ER proteins might be mediated through signaling mechanisms (Luedeke et al., 2005). Septin barrier is also responsible for reducing the movement of old nuclear pore complexes into the bud during cell division (Shcheprova, Baldi, Frei, Gonnet, & Barral, 2008).

Also mammalian septins are important for cell division. They have been shown to be involved in chromosome congression and segregation (Spiliotis, Kinoshita, & Nelson, 2005) and to regulate various steps of intercellular bridge formation and maturation, together with anillin, to ensure successful cytokinesis (Maddox, Lewellyn, Desai, & Oegema, 2007; Renshaw, Liu, Lavoie, & Wilde, 2014). Mammalian septins have several functions in addition to their role in mitosis. They regulate cell polarity, cytoskeletal organization, vesicle trafficking, ciliogenesis, and cell–pathogen interactions (Beites, Xie, Bowser, & Trimble, 1999; Ghossoub et al., 2013; Hsu et al., 1998; Hu et al., 2010; Kinoshita et al., 2002; Kremer, Adang, & Macara, 2007; Torraca & Mostowy, 2016; Zhai et al., 2014). In many of their functions, septins act as scaffolds, recruiting other proteins to the complex. For example, SEPT2 directly binds to nonmuscle myosin heavy chain IIA and regulates myosin activation by bringing myosin II and its kinases in close proximity (Joo, Surka, & Trimble, 2007), and the SEPT2/6/7 complex controls DNA damage response by interacting with the suppressor of cytokine signaling 7 (Kremer et al., 2007). In addition, septin scaffolds regulate exocytosis through interactions with syntaxin, an *N*‐ethylmaleimide–sensitive fusion protein attachment protein receptor (SNARE) (Beites et al., 1999). At the base of primary cilia, septins function as a diffusion barrier to ciliary membrane proteins that are essential for correct ciliary structure and function (Chih et al., 2011; Hu et al., 2010). The role of septins in ciliogenesis in kidney cells is reviewed in more detail below. Interestingly, in spermatozoa septins are found at the sperm annulus. The annulus is a ring‐like structure essential for the motility of sperm flagella and generation of the diffusion barrier that separates the midpiece and principal piece membrane domains. Septins are essential for forming this diffusion barrier (Ihara et al., 2005; Kwitny, Klaus, & Hunnicutt, 2010). Septins also form a ring at the base of dendritic spines in neurons, which is essential for dendrite branching and morphology (Xie et al., 2007). Human septin complexes also regulate bacterial entry into host cells by coordinating rearrangement of membrane–cytoskeletal structures. Moreover, septins associate with intracellular pathogens, by being recruited to the inclusion vacuoles in which the pathogens can replicate or assemble around bacteria that are capable of polymerizing actin, and thus regulate host defense mechanisms (Volceanov et al., 2014); reviewed in (Torraca & Mostowy, 2016).

## 2 STRUCTURE AND FUNCTION OF KIDNEY

**Figure 2:**
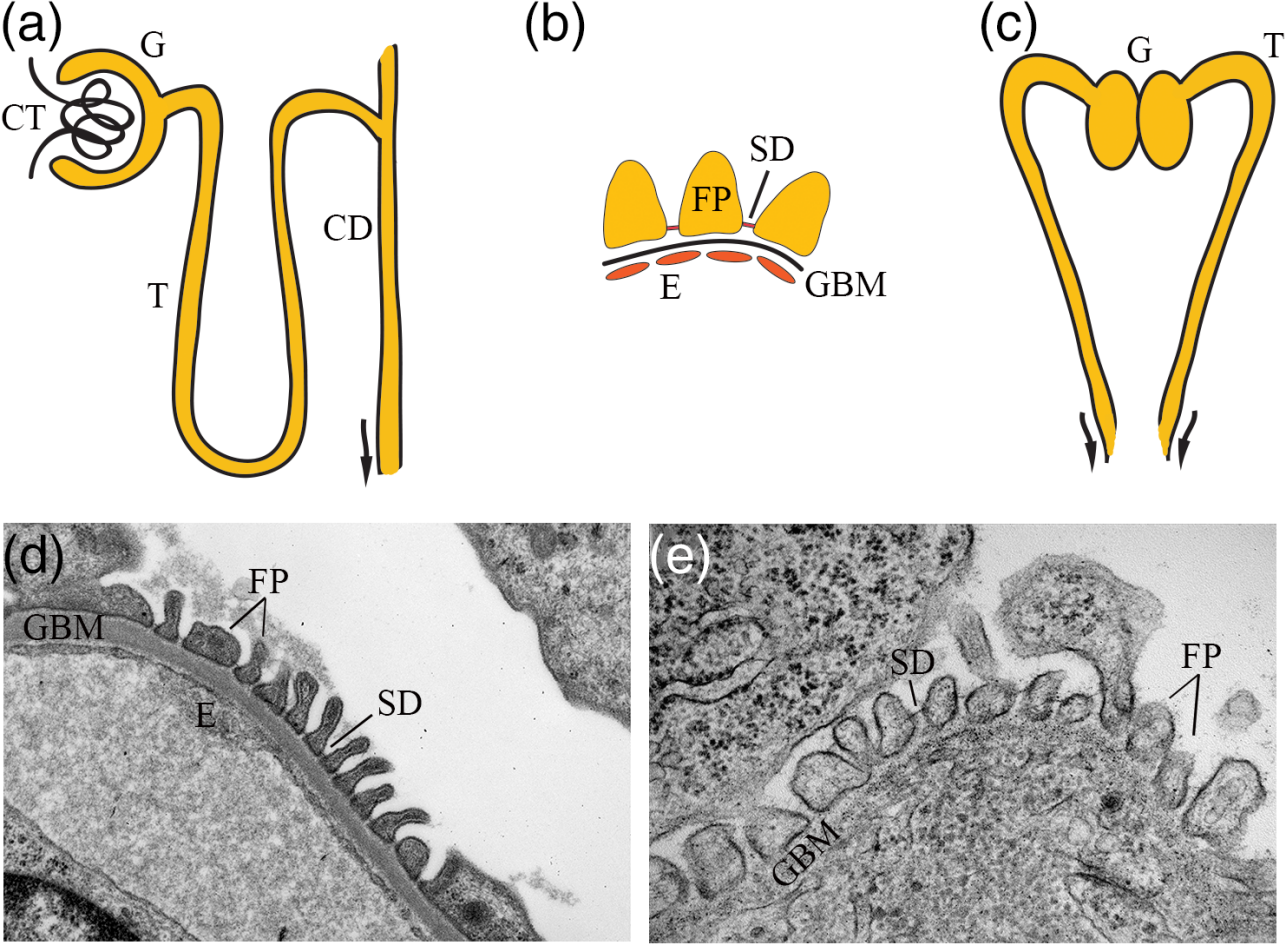
Schematic structure of mammalian nephron (a), glomerular filtration barrier (b) and zebrafish pronephros (c). Electron microscopic image of mouse (d) and zebrafish (e) glomerular filtration barrier visualizing their structural similarity. CD = collecting duct; CT = capillary tuft; E = endothelium; FP = podocyte foot process; G = glomerulus; GBM = glomerular basement membrane; SD = slit diaphragm; T = tubule [Color figure can be viewed at wileyonlinelibrary.com]

The human kidney contains approximately 1 million functional units or nephrons. The nephron consists of a glomerulus and a tubule, which can be divided into segments, the proximal tubule, the loop of Henle, and the distal tubule, which is connected to the collecting duct (Figure 2a). The ultrafiltration of plasma occurs in the glomeruli. The glomerular filtration barrier consists of the glomerular epithelial cells or podocytes, the glomerular basement membrane and the fenestrated endothelial cells (Figure 2b). Podocytes consist of the cell body and foot processes that enwrap the glomerular capillaries. Neighboring foot processes are interconnected with specialized cell adhesion structures called slit diaphragms (Figure 2b,d,e). The slit diaphragms, together with the fenestrated endothelial cells and the glomerular basement membrane, retain proteins of the size of albumin and larger in the circulation but allow water and small molecules to pass from the circulation into the glomerular ultrafiltrate. Water and essential molecules are then removed from the ultrafiltrate in the tubular system of the nephron. Upon injury, podocyte foot processes lose their structural characteristics (efface) and detach from the basement membrane, slit diaphragms are lost, and proteinuria develops (Shankland, 2006).

The pronephros in the larval zebrafish has two nephrons with pronephric tubules and a fused glomerulus in the midline (Figure 2c) (Drummond & Davidson, 2010). The cells that will develop to the pronephros arise from the ventral mesoderm and epithelialize by 24 h post‐fertilization (hpf). Patterning of the nephron into different segments, the pronephric tubule and the pronephric glomerulus, occurs between 24 and 40 hpf and the structure is vascularized at 40–48 hpf coinciding with the start of glomerular filtration. The nephron is fully mature with size‐selective glomerular filtration barrier at 4 days post‐fertilization (Drummond & Davidson, 2010). Despite of the great difference in the numbers of nephrons in the kidney, the mammalian and zebrafish glomerular filtration barriers share the same components and structure, including the podocytes, the glomerular basement membrane and the fenestrated endothelial cells as visualized by electron microscopy in Figure 2d,e. Also the tubular part of the nephron in zebrafish resembles its mammalian counterpart and can be divided into segments, including the neck, the proximal and distal tubules and the collecting duct (Drummond & Davidson, 2010). The shared structural features of the zebrafish pronephros and the mammalian permanent kidney, coupled with easy genetic manipulation and imaging of embryonic and larval zebrafish, make zebrafish an excellent model to study kidney organogenesis, define the role of different proteins in regulating kidney function and model and learn about kidney diseases.

## 3 EXPRESSION AND LOCALIZATION OF SEPTINS IN KIDNEY

**Figure 3:**
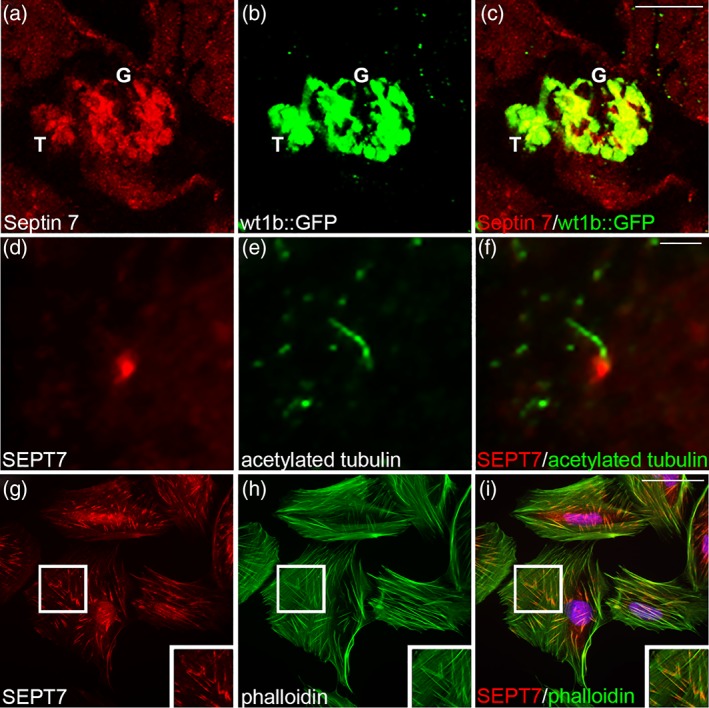
SEPT7 expression in the kidney. (a–c) Septin 7 localizes in the pronephric tubule cells (T) and the glomerulus (G) of 4‐dpf *wt1b::GFP* zebrafish larvae. The images are similar to figure 1J,K in Dash et al. (2014). (d–f) In IMCD3 cells, SEPT7 is expressed at the base of cilia, which are stained for acetylated tubulin. The images are similar to supporting information figure S3A in Dash et al. (2014). (g–i) In proliferating podocytes, SEPT7 appears as filaments which partially align along actin stress fibers labeled with phalloidin. The images are similar to supporting information figure S1A–D in Wasik et al. (2012). Scale bars: 40 μm (a–c); 2.5 μm (d–f); 20 μm (g–i) [Color figure can be viewed at wileyonlinelibrary.com]

Serial analyses of gene expression of septin mRNA expression in human tissues revealed the presence of SEPT1, SEPT2, SEPT7, SEPT8, and SEPT9 in the kidney (Cao et al., 2007). In mouse and zebrafish kidney, Septin 7 localizes to both glomeruli and tubules (shown for zebrafish in Figure 3a–c) (Dash et al., 2014; Wasik et al., 2012). In human kidney, SEPT2 has been shown to localize to glomeruli (Craven et al., 2006). In cultured inner medullary collecting duct (IMCD3) cells, SEPT2 and SEPT7 have been shown to localize at the base of the cilia (shown for SEPT7 in Figure 3d–f) as well as in a vesicular pattern in the cytoplasm (Dash et al., 2014; Hu et al., 2010). In epithelial MDCK cells, SEPT2 fibers colocalize with polyglutamylated microtubules near the trans‐Golgi network sites of apical and basolateral protein export (Spiliotis, Hunt, Hu, Kinoshita, & Nelson, 2008). In normal rat kidney (NRK) cells, SEPT2 partially colocalizes with actin on stress fibers and is also detected on intracellular vesicular structures (Schmidt & Nichols, 2004). In cultured podocytes, SEPT7 is expressed in both proliferating and differentiated cells (Wasik et al., 2012). In proliferating podocytes, SEPT7 localizes in the midbodies of cells undergoing cytokinesis, and in both proliferating and differentiated podocytes, it appears as filaments, partially localizing along actin stress fibers, and in a punctate pattern in the cytoplasm (Figure 3g–i) (Wasik et al., 2012). In addition, SEPT9 and SEPT11 have been detected in differentiated human podocytes and they form a complex with SEPT7 (Wasik et al., 2012).

## 4 FUNCTION OF SEPTINS IN KIDNEY

To date, knockout mice have been generated for 7 out of the 13 mouse septins. Of these, disruption of *Sept3* and *Sept6* cause little or no abnormalities (Fujishima, Kiyonari, Kurisu, Hirano, & Kengaku, 2007; Ono et al., 2005). However, disruption of *Sept7*, *Sept9*, and *Sept11* lead to embryonic lethality (Fuchtbauer et al., 2011; Menon et al., 2014; Roseler et al., 2011). *Sept7* ^−/−^mice are arrested early in development due to defective mitosis (Menon et al., 2014). *Sept9* ^−/−^ embryos die by Day 10 of gestation due to mesenchymal tissue degeneration and extensive cell death (Fuchtbauer et al., 2011) and *Sept11* ^−/−^ mice die by Day E13.5 (Roseler et al., 2011). *Sept4* ^−/−^ mice exhibit reduced sperm motility (Ihara et al., 2005; Kissel et al., 2005) and attenuation in dopaminergic transmission in neurons (Ihara et al., 2007). Loss of *Sept5* leads to various effects on specific affective behaviors and cognitive processes, and some of these changes depend on the genetic background of the mice (Suzuki et al., 2009). Kidney function has not been reported to be abnormal in any of the *Sept* knockout mice, but the embryonic lethality of *Sept7*, *Sept9,* and *Sept11* knockouts leaves open the role of these specific septins in the kidney. Thus far the functional studies of septins in kidney have mainly concentrated on using cultured kidney cells and zebrafish as models as described below.

### 4.1 Regulation of microtubule organization and epithelial morphogenesis

In MDCK cells, a well‐established model for studying renal epithelial morphogenesis, SEPT2 localizes with polyglutamylated tubulin during interphase and SEPT2 depletion leads to the loss of this subset of microtubules (Spiliotis et al., 2008). Moreover, the authors showed that SEPT2 is essential for the vesicle transport of membrane proteins from the Golgi to the plasma membrane along these polyglutamylated microtubules, as visualized by intracellular accumulation of apical and basolateral membrane markers in the absence of SEPT2 (Spiliotis et al., 2008). SEPT2 was shown to compete with microtubule‐associated protein 4 (MAP4) in binding to microtubules, as SEPT2/6/7 complexes can occupy the binding sites of MAP4 on tubulin and interfere with MAP4–tubulin binding (Spiliotis et al., 2008). As a consequence, cells depleted of SEPT2 lack vertically oriented polyglutamylated microtubules and fail to exhibit morphological characteristics of a polarized epithelium (Spiliotis et al., 2008). A later study revealed that SEPT2 is essential for the organization of perinuclear and peripheral microtubules and protects both from catastrophic depolymerization (Bowen, Hwang, Bai, Roy, & Spiliotis, 2011). Depletion of SEPT2 also altered the directional movement of the plus ends of the microtubules, reducing their apical positioning, and thereby affecting the development of apicobasal polarity of epithelial cells (Bowen et al., 2011). However, the role of SEPT2 in the microtubule regulation is cell‐type dependent. Microtubule organization was shown to be unaffected upon knocking down SEPT2 by siRNA, and the amount of tubulin in NRK epithelial cells was unchanged (Schmidt & Nichols, 2004). Instead, in these cells, septins play a role in regulating actin cytoskeleton as detailed below.

### 4.2 Regulation of cell motility

In NRK cells, SEPT2 is required for the maintenance of normal actin protein levels and global reorganization of the actin cytoskeleton. Interestingly, SEPT2 is not associated with actin in regions where highly dynamic actin is constantly remodeled, for example, at the leading edge of moving cells. SEPT2 partially colocalizes with actin stress fibers, thereby stabilizing the structures (Schmidt & Nichols, 2004). A recent study reported a novel network of septin filaments important for the organization of the lamellar stress fiber network, focal adhesion maturation and cell migration (Dolat et al., 2014). During normal development and tumor metastasis, epithelial cells undergo an epithelial‐to‐mesenchymal transition (EMT) that enhances cell motility. In kidney cells undergoing EMT, SEPT9 was shown to be upregulated, and knockdown of SEPT9 inhibited the maturation of focal adhesions and hindered cell motility (Dolat et al., 2014). The authors found that SEPT9 cross‐links preassembled actin stress fibers at the front of migrating kidney epithelial cells and thereby maintains the organization of the lamellar actin network (Dolat et al., 2014).

### 4.3 Regulation of exocytosis and glucose uptake

A protein secretion assay utilizing human embryonic kidney 293 (Hek293) cells revealed that siRNA‐mediated depletion of SEPT2 or impairment of the assembly and disassembly of septin hetero‐oligomers by forchlorfenuron (FCF) decrease the secretion of a model protein by reducing its post‐Golgi trafficking (Tokhtaeva et al., 2015). The authors also showed that FCF‐treatment of MDCK cells led to changes in the size and distribution of SEPT2 filaments and accumulation of cargo‐carrying intracellular vesicles destined to be trafficked to the plasma membrane, indicating that SEPT2 and dynamic reorganization of septin filaments are essential for exocytosis (Tokhtaeva et al., 2015). Several other studies point out a role for septins in vesicle trafficking, proposing also inhibitory effects for septins in exocytosis (Amin et al., 2008; Beites et al., 1999; Wasik et al., 2012). Interestingly, septins contribute in the regulation of membrane trafficking also in other cell types, including megakaryocytes and platelets (Bartsch et al., 2011; Dent et al., 2002). Thus, one can assume that the roles of septins in regulating vesicle trafficking vary in different cell types, possibly depending on which septins are in complex in each specific cell type.

In proliferating podocytes, SEPT7 localizes in the midbody, and therefore apparently participates in the regulation of cell division (Wasik et al., 2012). However, mature podocytes in vivo are terminally differentiated and do not divide, suggesting another function for septins in differentiated podocytes. Consequently, disrupting the dynamic organization of septins by FCF‐inhibited glucose uptake into podocytes in both basal conditions and after insulin stimulation, suggesting that septins affect both the constitutive glucose transporter GLUT1 and the insulin‐stimulated glucose transporter GLUT4 (Wasik et al., 2012). The trafficking of GLUT4 storage vesicles (GSVs) involves a SNARE complex that facilitates the docking and fusion of the GSVs with the plasma membrane (reviewed in (Jaldin‐Fincati, Pavarotti, Frendo‐Cumbo, Bilan, & Klip, 2017). The SNARE complex includes a v‐SNARE on GSVs, vesicle‐associated membrane protein 2 (VAMP2), and t‐SNAREs on the plasma membrane, such as syntaxin 4 and SNAP23 (Jaldin‐Fincati et al., 2017). SEPT7 was found to interact with VAMP2 and nephrin (Wasik et al., 2012), a plasma membrane protein that also interacts with VAMP2 and thereby enhances the fusion of the GSVs with the plasma membrane (Coward et al., 2007). As depletion of SEPT7 was found to increase the interaction of nephrin with VAMP2, SEPT7 was proposed to hinder GSV trafficking by forming a physical barrier between the vesicles and the plasma membrane by associating with v‐SNAREs and nephrin (Wasik et al., 2012). Moreover, SEPT7 also binds to the t‐SNARE SNAP23 suggesting that SEPT7 could be involved in the regulation of the GSV docking process (Wasik et al., 2012). In line with this, SEPT7 was found to form a complex with nonmuscle myosin heavy chain IIA (NMHC‐IIA; encoded by *MYH9*; Wasik et al., 2017), which has been shown to regulate glucose uptake in adipocytes by influencing the docking and fusion of GSVs as well as the activity of GLUT4 (Steimle, Fulcher, & Patel, 2005). NMHC‐IIA was shown to form a complex with SNAP23, and SEPT7 reduced the activity of NMHC‐IIA in this complex thereby reducing the docking and fusion of GSVs with the plasma membrane (Wasik et al., 2012).

### 4.4 Ciliogenesis in vitro

An interesting discovery in cultured IMCD3 cells was the localization of SEPT2 at the base of the primary cilium (Chih et al., 2011; Hu et al., 2010), resembling the localization of septins at the mother–bud neck in budding yeast forming a diffusion barrier (Haarer & Pringle, 1987; Hartwell, 1971). The primary cilia in mammalian kidney epithelial cells have been proposed to be non‐motile sensory organelles. Primary cilium is a microtubule‐based antenna‐like organelle that projects from the epithelial apical membrane into the lumen of the nephron tubule (Pazour & Witman, 2003). The function of the primary cilium is to sense the flow of fluid in the tubular lumen and to control signaling pathways. Depletion of SEPT2 in the IMCD3 cells resulted in complete loss of primary cilia, and partial loss led to their shortening (Hu et al., 2010). Similar to yeast, SEPT2 was shown to be part of a membrane diffusion barrier in IMCD3 cells, maintaining the localization of ciliary membrane proteins. Removal of this diffusion barrier by depletion of SEPT2 led to defective Sonic hedgehog signaling and inhibition of ciliogenesis (Hu et al., 2010). In addition to localizing at the base of cilia, SEPT2 was also observed along and at the tip of the axoneme in a proportion of IMCD3 cells (Hu et al., 2010). Another study carried out with retinal pigment epithelial cells showed that SEPT2 forms a complex with SEPT7 and SEPT9, and that the complex localizes along the axoneme, accumulating in cilium upon its maturation, as well as along cytoplasmic fibers colocalizing with actin filaments (Ghossoub et al., 2013). The complex was shown to control ciliogenesis and the length of cilia (Ghossoub et al., 2013). In ciliated respiratory epithelial cells, septins are expressed in the cytoplasm as well as at the ciliary base (SEPT2, SEPT4, SEPT4, and SEPT7), along the axoneme (SEPT2, SEPT7, SEPT9, and SEPT11) or in specific ciliary sub‐compartments (SEPT8 and SEPT9) (Fliegauf, Kahle, Haffner, & Zieger, 2014). The data thus indicate that the localization of septins varies, suggesting distinct functions and apparently also distinct regulatory mechanisms depending on the cell type.

### 4.5 Ciliogenesis and pronephric fluid flow

**Figure 4:**
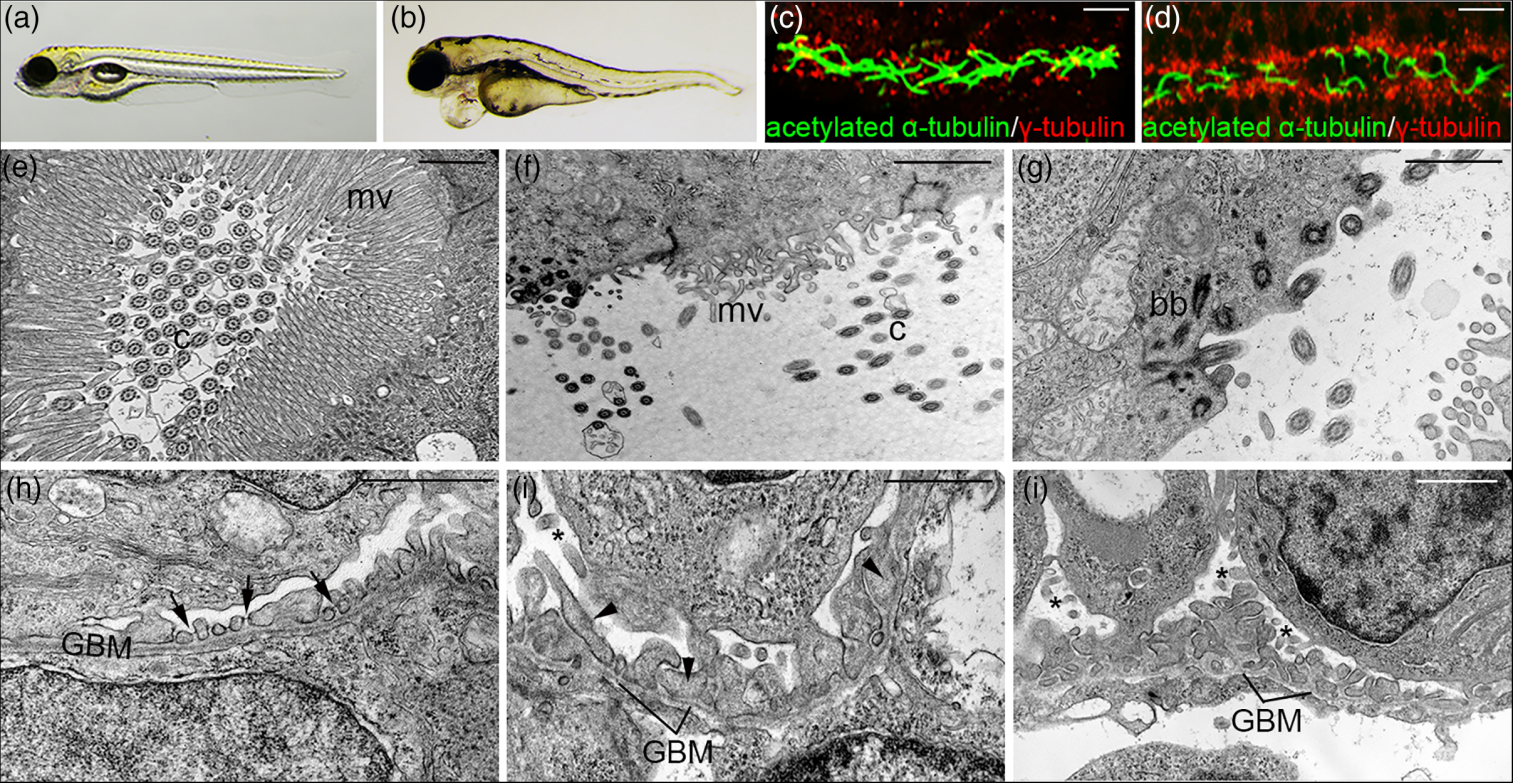
Knockdown of *sept7b* causes body curvature, edema, and defects in the structure of the pronephros. (a,b) Knockdown of *sept7b* causes pericardial and yolk sac edema at 4 dpf (b), whereas the control larva does not show phenotypic abnormalities (a). The images are similar to figure 5G,I in Dash et al. (2014). (c) Staining of cilia using antibodies to acetylated α‐tubulin and basal bodies using antibodies to γ‐tubulin in the pronephric tubule in control embryos shows well‐organized basal bodies that localize at the base of cilia in the tubular epithelial cells. (d) in *sept7b* knockdown larva, the basal bodies are disorganized, the tubule diameter is increased and cilia appear misoriented. The images in c and d are similar to figure 5G‐H in Dash et al. (2014). (e) a pronephric tubule of the wild‐type larva shows regular microvilli and cilia morphology. (f,g) A tubule in a s*ept7b* morphant appears dilated and shows sparse and irregular microvilli and scattered cilia. (h) Wild‐type podocytes show regular foot processes connected by slit diaphragms (arrows). (i,j) Podocytes of a s*ept7b* morphant show foot process effacement (arrowhead) and microvillar‐type cell processes (asterisk) on their apical surface. The images in (e–j) are similar to Figure 3a–e in Dash et al. (2014). bb = basal body; c = cilia; GBM = glomerular basement membrane; mv = microvilli. Scale bars: 20 μm, (c,d); 1 μm [Color figure can be viewed at wileyonlinelibrary.com]

Cilia in the zebrafish larval kidney are motile and disruption of their structure or function results in loss of fluid flow, accumulation of fluid and pronephric cyst formation (Kramer‐Zucker et al., 2005). Thus far the functional role of two septins, *sept6* and *sept7b*, orthologs of human *SEPT6* and *SEPT7*, have been addressed in zebrafish pronephric kidney. *sept6* mRNA and Septin 7 protein were found to be expressed in the pronephros (shown for Septin 7 in Figure 3a–c) and other ciliated organs in larval zebrafish (Dash et al., 2014; Zhai et al., 2014). Confocal microscopy analysis indicated the presence of Septin 7 in both the cytoplasm and at the base and along the cilia in pronephric duct cells (Dash et al., 2014). Morpholino antisense oligonucleotide‐mediated knockdown of either *sept6* or *sept7b* led to body axis curvature and also pericardial edema and pronephric cyst formation (shown for *sept7*b in Figure 4a,b), both signs of defective pronephric function (Drummond et al., 1998). Furthermore, both knockdowns led to reduced number and length of pronephric cilia (Dash et al., 2014; Zhai et al., 2014). Depletion of *sept7b* caused misorientation of basal bodies and cilia in the pronephric tubules (Figure 4c–g) without affecting the normal 9 + 2 organization of the microtubules in the axoneme of the cilia (Dash et al., 2014). This led to aberrant and slow beating of the cilia and defects in fluid flow, reducing the kidney function (Dash et al., 2014).

In search for the mechanisms leading to defective ciliogenesis, Septin 6 was found to form a complex with acetylated alpha‐tubulin in zebrafish larvae, and the authors proposed this as a mechanism by which Septin 6 regulates ciliogenesis (Zhai et al., 2014). The study on *sept7b* addressed both regulation of actin and vesicle trafficking as potential mechanisms by which *sep7b* controls organization of basal bodies and ciliogenesis in pronephros. Actin accumulation was reduced in the apical domain of pronephric tubule cells upon *sept7b* depletion (Dash et al., 2014), in line with previous studies in cultured cells showing that depletion of septins destabilizes actin (Kinoshita et al., 2002). This may affect the migration and docking of basal bodies to the apical plasma membrane (Pan, You, Huang, & Brody, 2007; Ravanelli & Klingensmith, 2011). The study further revealed that in IMCD3 cells, SEPT7 partially colocalizes and forms a complex with the small GTPase Rab8 (Dash et al., 2014), known to regulate vesicle trafficking and ciliogenesis (Nachury et al., 2007; Yoshimura, Egerer, Fuchs, Haas, & Barr, 2007). In *Xenopus* embryos Septin 7 regulates ciliogenesis together with planar cell polarity (PCP) protein Fritz (*wdpcp*) (Kim et al., 2010). *Wdpcp*‐deficient mice exhibit phenotypes reminiscent of ciliopathy syndromes and *Wdpcp*‐deficient cells fail to recruit proteins required for ciliogenesis, including Septin 2, to the ciliary transition zone (Cui et al., 2013). Defining whether the PCP pathway together with *sept7b* is involved in regulating ciliogenesis also in the zebrafish requires further studies.

In addition to defects in the ciliary function in the pronephros, zebrafish larvae depleted of *sept7b* also present with ciliary defects in the Kupffer's vesicle and the central canal, giving rise to defects in the left–right asymmetry and hydrocephaly. These results indicate that *sept7b* plays an important role in the structural and functional organization of the pronephros and a general role in regulating the structure and function of motile and non‐motile cilia. Notably, loss of *sept7b* expression gives rise to multiple developmental defects that are similar to human ciliopathies. Similarly, knockdown of *sept6*, in addition to causing pronephric cysts, also results in laterality defects (Zhai et al., 2014). Thus far no septin complexes have been purified from zebrafish, but the similarity of the phenotypes caused by knockdown of *sept6* and *sept7b* raises a question whether these septins could form a complex and function together in zebrafish, in the same way as SEPT2, 6, and 7 have been shown to form a complex in mammalian cells (Joberty et al., 2001; Kinoshita et al., 2002). Staining of adult human kidney tissue sections revealed that SEPT9 colocalizes with acetylated tubulin in the cilia further supporting a role for septins in regulating ciliogenesis and ciliary function also in higher vertebrates (Ghossoub et al., 2013).

### 4.6 Glomerular ultrafiltration

In addition to pronephric tubules, Septin 7 protein is also expressed in podocytes in zebrafish (Figure 3a–c) (Dash et al., 2014). Knockdown studies further revealed that *sept7b* knockdown affects the integrity of the glomerular filtration apparatus, demonstrated by leakage of high‐molecular weight substances through the barrier into the glomerular filtrate in larvae lacking *sept7b* (Dash et al., 2014). Accordingly, podocytes showed morphological changes, including effacement of podocyte foot processes and extension of microvillar‐type projections (Figure 4h–j) (Dash et al., 2014). However, whether lack of *sep7b* affects directly podocyte differentiation or whether the glomerular filtration defect is due to reduced fluid flow and the consequently elevated fluid pressure, or both, requires further studies.

## 5 SEPTINS AND KIDNEY DISEASES

Septins have been implicated in kidney cancer. SEPT2 and SEPT11 are upregulated in renal cell carcinoma (RCC), an aggressive and highly metastatic cancer of the kidney with poor response to therapeutics (Craven et al., 2006; Craven, Stanley, et al., 2006). The von Hippel Lindau (VHL) tumour suppressor gene plays a central role in development of RCCs and interestingly, increased SEPT2 expression in RCC was shown to be VHL‐dependent (Craven, Hanrahan, et al., 2006).

The knockdown of *sept7b* in zebrafish larvae results in pronephric tubular cysts and expansion of the Bowman's space resembling the phenotypes after knocking down of genes involved in ciliogenesis and/or associated with polycystic kidney disease (Obara et al., 2006; Sullivan‐Brown et al., 2008). However, thus far no mutations in septins have been reported in human patients with cystic kidney diseases. Yet, the role for septins in cilia‐associated disorders has been speculated (Barral, 2010). This dates back to the finding that in *Xenopus*, the PCP protein Friz (Wdpcp) associates with Septin 2 and the localization of both Septin 2 and Septin 7 is disturbed in Fritz morphants (Kim et al., 2010). Mutations in human Fritz, in turn, were identified in Meckel‐Gruber and Bardet‐Biedl syndromes, human ciliopathic disorders characterized also by renal abnormalities (Hildebrandt, Benzing, & Katsanis, 2011). A recent study identified SEPT2 as an interaction partner of DAZ interacting protein 1‐like (DZIP1L), encoded by *DZIP1L*, which is mutated in autosomal recessive polycystic kidney disease (Lu et al., 2017). Time will show whether mutations in septin genes are identified in ciliopathies or whether septins contribute to the development of these disorders via interactions with proteins known to be associated with ciliopathies.

## 6 CONCLUSIONS AND PERSPECTIVES

The past 20 years have seen considerable progress in understanding the mechanisms via which septins regulate various cellular processes, yet they have remained poorly characterized in the kidney. Septins have been shown to form hetero‐oligomeric complexes in kidney cells in culture and those complexes show cell‐dependent functions, including control of vesicle trafficking, glucose uptake, and regulation of actin cytoskeleton and motility. SEPT9 has been linked to EMT, a process associated with tumor metastasis, in kidney cells in culture, and the expression of SEPT2 and SEPT11 is increased in RCC. However, it is unclear whether and via which mechanism septin upregulation would contribute to RCC growth and metastasis. Further work is also needed to define whether septins could be used for kidney disease diagnostics and potentially also as targets for treatment.

Septins are essential regulators of ciliogenesis as depletion of septins results in loss of cilia both in vitro and in vivo. In zebrafish, the phenotype of septin knockdown resembles the knockdown phenotypes of genes involved in ciliopathies. Despite of the functional evidence linking septins to ciliogenesis in zebrafish, many questions remain to be answered about their roles in this process and ciliopathies in general. Of great interest will be searches for mutations in septins in ciliopathies, or potential identification of septins as part of protein interaction networks associated with the development of ciliary diseases.

Also understanding how the dysregulation of the function of septins potentially associates with renal glomerular diseases in higher vertebrates is an exciting area of future research. For this, a deeper understanding of the expression of the different septins, their complex formation and interactions with other proteins that are necessary for glomerular functions is essential. In the case of the septin knockout mice with embryonic lethal phenotypes, podocyte‐specific knockout mouse models are necessary for defining the role of these specific septins in the maintenance of glomerular ultrafiltration in higher vertebrates. A recent study revealed that septin network is required for the process of mechanotransduction (Calvo et al., 2015). Mechanical stimuli, including stretch, affect podocyte biology in vitro and play a significant role in the development of glomerulosclerosis in vivo (Schordan et al., 2013). Thus, it would be interesting to see how the absence of septins in podocytes affects glomerular function in experimentally induced conditions that create mechanical forces arising from increased glomerular capillary pressure.
